# Editorial Comment: Robotic surgery using Senhance® robotic platform: single center experience with first 100 cases

**DOI:** 10.1590/S1677-5538.IBJU.2020.04.07

**Published:** 2020-05-20

**Authors:** Eliney F. Faria

**Affiliations:** 1 Serviço de Urologia Hospital Felicio Rocho Belo HorizonteMG Brasil Serviço de Urologia, Hospital Felicio Rocho, Belo Horizonte, MG, Brasil

Samalavicius NE^1,2^, Janusonis V^3,4^, Siaulys R^3^, Jasenas M^3^, Deduchovas O^3^, Venckus R^3^, Ezerskiene V^3^, Paskeviciute R^3^, Klimaviciute G^3^

^1^ Department of Surgery, Klaipeda University Hospital, 41 Liepojos Str., 92288, Klaipeda, Lithuania; ^2^ Clinic of Internal, Family Medicine and Oncology, Faculty of Medicine, Vilnius University, ^2^ Santariskiu Str., 08660, Vilnius, Lithuania; ^3^ Department of Surgery, Klaipeda University Hospital, 41 Liepojos Str., 92288, Klaipeda, Lithuania; ^4^Faculty of Health Sciences, Klaipeda University, 84 H. Manto Str., 92294, Klaipeda, Lithuania

J Robot Surg. 2019 Jul 12. [Epub ahead of print]

**DOI: 10.1007/s11701-019-01000-6 | ACCESS: 10.1007/s11701-019-01000-6**

## COMMENT

In this paper Dr Samalavicius, reported that robotic surgery today has already had a long tradition and use only option for performing robotic surgery (da Vinci robotic system), which has been the for almost past two decades. This paper describe a cohort using the Senhance® robotic system (TransEnterix Surgical Inc., Morrisville, NC, USA). In contrast to a previous existing robotic platform. This novel system has haptic feedback and the camera can be operated with an “eye-sensing control”. After a successful cohort in gynecology and colorectal surgery. This system is approved in Europe and USA and pronounce lower costs per operation. This system uses standard surgical trocars and can be positioned in the typical laparoscopic positions for the different interventions. All surgeries included in their article were performed from November 2018 to March 2019 a total of 100 procedures using the Senhance® robotic platform in general and colorectal surgery, gynecology, and urology (31 procedures, of them 27 radical prostatectomies). There were 3 (3%) conversions: two to laparoscopy (both undergoing robotic radical prostatectomy) and one to open (undergoing total hysterectomy). The reasons for conversions to laparoscopy were technical difficulties for continuing with robotic surgery due to difficult pelvic anatomy, and unexpected findings for conversion to open surgery. Complication rate was reasonable and occurred in 16 patients (35.5%), but only 2 (4.4%) complications were severe (Clavien–Dindo III); none of his patients demanded reoperation. The authors reported their experience in radical prostatectomies using this system is the first in literature. They clarified that more detailed analysis about radical robotic prostatectomies will be published separated in near future paper. They concluded the experience with different types of robotic surgeries allows them to state that the Senhance® robotic system is feasible and safe for general surgery, gynecology, and urology. They believe a wider implementation of this system worldwide is simply a question of time.

